# Weight-for-length relationship at birth to predict neonatal diseases

**DOI:** 10.1590/S1516-31802003000400002

**Published:** 2003-07-01

**Authors:** José Ricardo Dias Bertagnon, Conceição Aparecida de Mattos Segre, Gloria Maria Dall Colletto

**Keywords:** Newborn, Weight, Crown-rump length, Body mass index, Recém-nascido, Peso, Comprimento, Índice de massa corporal

## Abstract

**CONTEXT:**

Intrauterine growth curves are extremely useful for classifying newborn children and predicting neonatal diseases. However, such curves rely on knowledge of the gestational age, which is not always easily obtained. Therefore, the study of other anthropometric measurements and their interrelationship is always desirable, in order to attain such objectives.

**OBJECTIVE:**

To evaluate whether newborns’ birth weight and length can identify neonatal diseases, independent of knowledge of the gestational age.

**TYPE OF STUDY:**

Retrospective study.

**SETTING:**

Institute of Teaching and Research of Hospital Israelita Albert Einstein, São Paulo, Brazil.

**PARTICIPANTS:**

During the period from February 1995 to January 1998, 8,397 live newborns were studied in the hospital's maternity ward.

**PROCEDURES:**

The weight and length of live newborns were obtained at birth, thus allowing the analysis of weight-for-length adequacy, i.e. the distribution of birth weight for each class of birth lenght. These measurements were determined for the first 4,634 live newborns and the 10th and 90th percentiles were established. These parameters were applied to the next 3,763 consecutive newborns of the same population. The relationships between these variables and some neonatal diseases were investigated. The significance level adopted was p < 0.05.

**MAIN MEASUREMENTS:**

Birth weight and length, weight-for-length adequacy (10th and 90th percentiles for weight distribution in each 1-cm length class), weight/length index (10th and 90th percentiles of newborn's weight divided by the length) and frequent neonatal diseases in this population.

**RESULTS:**

There was a significant association of adequacy and index with the following affections: asphyxia, jaundice, hypoglycemia, hypomagnesemia, congenital pneumonia, pulmonary hypertension and sepsis. Additionally, there was a relationship between the index and respiratory distress syndrome, transient tachypnea and persistent ductus arteriosus.

**CONCLUSIONS:**

Weight-for-length adequacy and weight/length index alone, without the knowledge of gestational age, were able to identify newborns at risk for some selected neonatal diseases.

## RESUMO

**CONTEXTO:**

As curvas de crescimento intrauterino têm sido extremamente úteis para a classificação de recém-nascido, bem como para predizer doenças neonatais. Contudo, dependem do conhecimento da idade gestacional, que nem sempre é facilmente obtida. Assim, o estudo de outras medidas antropométricas, e suas inter-relações, tem sido proposto para as mesmas finalidades.

**OBJETIVO:**

Estabelecer a utilidade das medidas de peso e comprimento ao nascer na identificação de algumas doenças, independentemente do conhecimento da idade gestacional.

**TIPO DE ESTUDO:**

Estudo retrospectivo.

**LOCAL:**

Instituto de Ensino e Pesquisa Albert Einstein, São Paulo, Brasil.

**PARTICIPANTES:**

Foram estudados 8.397 nascidos vivos na maternidade do Hospital Israelita Albert Einstein, no período compreendido entre fevereiro de 1995 e janeiro de 1998.

**PROCEDIMENTOS:**

Peso e comprimento dos recém-nascidos, obtidos ao nascimento, permitiram o estudo da adequação do peso por classes do comprimento e o índice peso/comprimento. Foram calculados os percentis 10 e 90. Os parâmetros obtidos entre os primeiros 4.634 recém-nascidos foram aplicados aos sucessivos 3.763 nascidos vivos. Os recém-nascidos foram classificados em pequenos, adequados ou grandes, conforme estivessem abaixo, entre ou acima dos percentis 10 e 90. Foram pesquisadas associações dessas variáveis com algumas afecções neonatais, pelo teste do qui quadrado (p < 0,05).

**VARIÁVEIS ESTUDADAS:**

Peso e comprimento ao nascimento, adequação do peso por classes do comprimento, índice peso/comprimento, e afecções neonatais mais freqüentes nessa população.

**RESULTADOS:**

A adequação e índice mostraram-se significativamente associados às seguintes afecções: asfixia, icterícia, huipoglicemia, hipomagnesemia, pneumonia congênita, hipertensão pulmonar e sepse. O índice ainda se relacionou à síndrome do desconforto respiratório, taquipnéia transitória e persistência do canal arterial.

**CONCLUSÕES:**

As medidas de peso e comprimento ao nascer são facilmente obtidas e podem discriminar recém-nascidos de risco para algumas das principais morbidades neonatais, independentemente do conhecimento da idade gestacional.

## INTRODUCTION

Fetal growth may at any time during the gestational period be affected by several factors that can cause significant or minimal losses to the fetus. Maternal or environmental restrictive factors acting early in fetal life may damage the final cell population. On the other hand, any aggravating circumstance acting late in the gestational period may reduce the final fetus size. Accordingly, these factors may cause diseases to either fetus or newborn and thus interfere with neonatal morbidity and mortality.^1^

There are several methods for evaluating intrauterine growth. The curves relating birth weight to gestational age are valuable tools and classify the newborns as small, appropriate or large for gestational age if below the 10^th^ percentile, between the 10^th^ and 90^th^ percentiles or above the 90^th^ percentile of the distribution, respectively. They also correlate neonatal morbidity and mortality.^2^ These intrauterine growth curves have undoubtedly been most valuable for identifying newborns at risk, thereby allowing better allocation of funds and optimization of appropriate resources^1,2^. These curves, however, are directly dependent on knowledge of the gestational age, which is sometimes difficult to obtain.^3^

Several newborn anthropometric relationships have also been associated with morbidity and mortality, such as weight/length index, ponderal index, body mass index, cephalic perimeter/thoracic perimeter, expected weight/actual weight and weight/placental weight, among others.^4,5^ These parameters could also be used for identifying newborns at risk provided they are non-invasive and precise and have low cost.

The relationship between weight and length as a means of nutritional evaluation is based on the fact that biologically there seems to be greater protection for length increases than for weight increase. In 1989, Kramer et al.^6^ verified that full term undernourished infants had weight losses of up to 31% but length decreases of only 6%. As fat and muscle mass accumulate up to the end of the gestational period, it can easily be understood that the relationship between weight and length increases with gestational age.^7^ Therefore, the establishment of a curve that takes into account weight and length could translate any abnormalities in fetal growth, and this would be independent of prior knowledge of the gestational age.

Thus, the purpose of the present study was to evaluate the usefulness of weight and length measurements taken at birth, to identify some perinatal diseases selected on the basis of their frequency and importance among a population of high socioeconomic level.

## Methods

Based on a newborn population consisting of 9,675 consecutive live births in the maternity ward of Hospital Israelita Albert Einstein, São Paulo, Brazil, from 1995 to January 1998, a retrospective study was conducted to evaluate these newborns’ weight and length data at birth. This hospital unit provides maternal care for pregnant patients referred by private obstetricians who are not hospital employees but are duly authorized to use the hospital facilities.

The main characteristics of the mothers have already been described in a previous report.^8^ These women were an average of 31.1 years old, 61.1% were university graduates and 88.25% married, with an average of 2 previous pregnancies and 10 prenatal consultations. They were representative of a high socioeconomic group.

Data was gathered from 9,675 children and two samples were designated: sample 1, composed of the first consecutive 5,000 newborns, and sample 2, including the remaining 4,675 records. All twins and those newborns whose data on gestational age, sex, birth weight or length were lacking, and those presenting length damage due to congenital malformations, were excluded. Therefore, sample 1 consisted of 4,634 newborns and sample 2 was made up of 3,763 newborns.

Sample 1 was used for calculating and determining newborns’ weight-for-length adequacy parameters and weight/length index, with the respective 10^th^ and 90^th^ percentiles. Sample 2 was used for applying the above parameters and verifying any associations of weight-for-length adequacy and weight-forlength index with some neonatal diseases selected by frequency or importance among the population sample. This approach was chosen in order to avoid methodological bias.

All information was taken from the data bank that was available in the neonatal unit at the time of the study. It was gathered from the information registered in perinatal records that was entered by the obstetric nursing team and/or neonatologist attending the delivery. The information was compiled using the EPI-Info 6.04 program.

The following data were studied: 1) gestational age, based on the mother's obstetric history and calculated from the first day of her last normal period using Naegele's rule, converting the number of days into full weeks. This approach was adopted because all the pregnant women had prenatal care and were able to inform about their menstrual history. 2) Weight, which was obtained soon after birth by using a digital electric balance for newborns of capacity 10 kg and sensitivity of 5 grams; and length, using a wood anthropometric linear ruler with one wedge fixed and one sliding caliper, recording in centimeters to one decimal place.^9^ The staff neonatologist took this measurement within four to six hours of the birth. Lengths measured by different staff neonatologists were considered to be valid, because of a previous test performed between these measurements and those taken by only one researcher (JRDL) which showed r = 0.94 (95% CI of 0.84-0.91)^10^. 3) Data related to neonatal diagnoses, which was based on clinical and laboratory criteria, as indicated by protocols adopted at the neonatal unit during the study period.

The weight-for-length relationship was established in two ways: weight-for-length adequacy and weight-for-length index, both calculated from sample 1 data.

For the purposes of adequacy calculations, the lengths were listed and grouped into 1cm classes, thereby transforming this continuous variable into a discrete variable. For each length class, the weight distribution was checked with the identification of the 10^th^ and 90^th^ percentiles. These two cutoff points, the 10^th^ and 90^th^ percentiles, gave rise to the following newborn classification: "small for length" for newborns below the 10^th^ percentile; "appropriate for length" for newborns between the 10^th^ and 90^th^ percentiles, inclusive; and "large for length" for newborns over the 90^th^ percentile.

For the weight-for-length index calculation, the quotient of each newborn's weight divided by the length was used. This was done for all newborns in sample 1. The 10^th^ and 90^th^ percentiles were also calculated. The newborns were then classified as "small indexfor-length", "appropriate index-for-length" and "large index-for-length" according to their positioning below the 10^th^ percentile, between the 10^th^ and 90^th^ percentiles (inclusive) and over the 90^th^ percentile, respectively.

The newborns were also classified according to their gestational age (under or over 37 weeks, calculated according to Naegele's rule) with regard to adequacy and index.

In sample 2, the associations between adequacy and weight-for-length index (considered as independent variables) and variables related to some neonatal diseases were studied. The most frequent and important diseases considered were asphyxia (light, mild and severe, according to one-minute Apgar scores of 0-3, 46 and 7-10, respectively), physiological jaundice, respiratory distress syndrome (hyaline membrane disease of the newborn), transient tachypnea, congenital pneumonia, pulmonary hypertension, sepsis, hypoglycemia, hypomagnesemia, congenital dislocation of the hip, persistent ductus arteriosus, pneumomediastinum, pneumothorax and birth trauma. All of these were taken as dependent variables.

The statistical methodology considered arithmetical means, standard deviation and the minimum and maximum values used for describing samples. The accumulated frequency was used for calculating weight-for-length percentile distributions and index distributions. Linear regression and correlation coefficient and their significance were used for checking weight/length correlations. Samples were compared using the Mann-Whitney tests. All the aforementioned statistical tests were processed using the Statistical Package for Social Sciences (SPSS) V9.0 software (2000).

The present study was approved by the Research Ethics Committee of the Institution. Since all data were obtained from medical records, patients’ prior formal consent was not necessary.

## RESULTS

Comparison between samples 1 and 2 according to the Mann-Whitney test showed that maternal age and newborn weight were significantly higher in sample 2 and that gestational age and newborn length were similar in the two samples, as can be seen in Table 1.

**Table 1:** Mean and standard deviation (SD) of the characteristics of samples 1 (n = 4,634) and 2 (n = 3,763) and their respective Mann-Whitney tests (Z) and probabilities (P) for comparison between the two samples of newborns

Characteristic	Sample	Mean	SD	Minimum	Maximum	P	Z
Maternal age (years)	1	31.0	4.7	16	49	0.001	- 3.33
	2	31.3	4.5	11	49		
Gestational age (weeks)	1	38.7	1.7	23	42	0.119	- 1.56
	2	38.7	1.5	22	42		
Birth weight (g)	1	3215.4	488.5	610	5050	0.007	- 2.70
	2	3243.9	473.4	500	5250		
Length (cm)	1	49.3	2.2	30	55.5	0.131	- 1.51
	2	49.3	2.1	31.5	56.5		

Table 2 shows the 10^th^ and 90^th^ percentiles of the weight distribution for each length in sample 1 and their adjusted values. The best-fit values for adjusting these data were obtained by quadratic regression analysis, represented by the equations below:

P90 = −3,767 + 89.11 x length + 1.237 x length^2^ P10 = −9,490 + 306.5 x length – 1.113 x length^2^

**Table 2:** Values of the 10^th^ and 90^th^ percentiles of the birth weight-for-length distribution and their adjusted values in 4,634 newborns

Length (cm)	90^th^ Percentile	10^th^ Percentile	90^th^ Percentile Adjusted	10^th^ Percentile Adjusted
40	1,687	999	1,777	989
41	1,835	1,220	1,967	1,206
42	2,241	1,607	2,158	1,420
43	2,530	1,357	2,353	1,632
44	2,570	1,720	2,549	1,841
45	2,832	2,097	2,749	2,049
46	2,960	2,310	2,950	2,254
47	3,180	2,550	3,155	2,457
48	3,330	2,710	3,361	2,658
49	3,530	2,883	3,570	2,856
50	3,727	3,060	3,782	3,053
51	3,914	3,201	3,996	3,247
52	4,180	3,440	4,213	4,339
53	4,320	3,556	4,432	3,628
54	4,710	3,729	4,653	3,816
55	5,000	4,110	4,877	4,001

**Figure 1:**
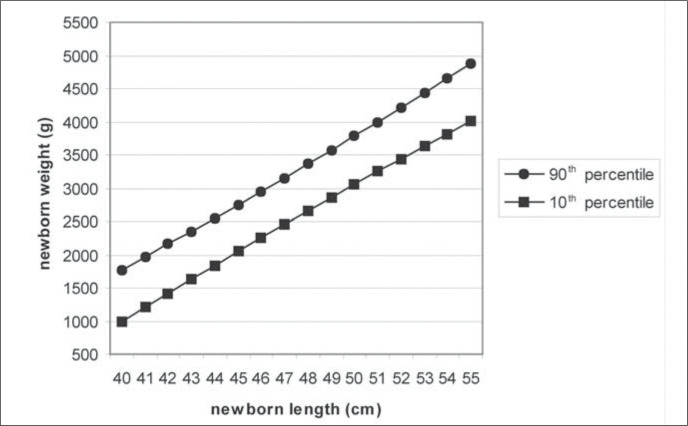
Regression lines for weight/length distribution at birth for the 10th and 90th percentiles.

**Figure 2:**
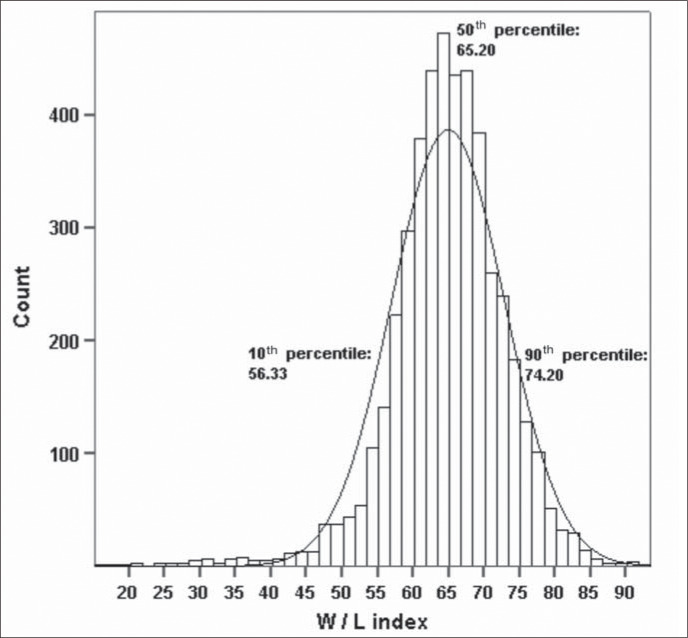
Histogram frequency of the weight/length (W/L) index.

Figure 1 depicts the adjusted data for weight and length and Figure 2 shows the graphical representation of the index distribution with the values of the 10^th^, 50^th^ and 90^th^ percentiles for that distribution. Considering all the newborns, 10% had a weightfor-length of less than 56.33 and 10% had more than 74.20.

Among the newborns classified by the weight-for-length index, the preterm proportions were 43.2, 3.1 and 0.9% for small, appropriate and large index-for-length categories, respectively. However, for newborns classified by adequacy, the proportions of preterm infants were 13.1, 6.6 and 3.1%, for the small, appropriate and large-for-length categories, respectively.

**Figure 3:**
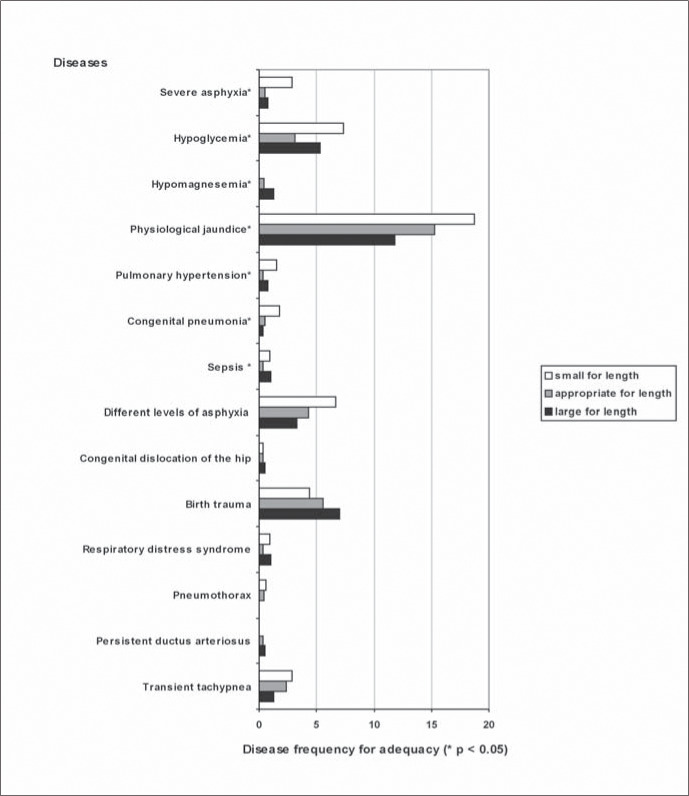
Neonatal disease frequency and weight-for-length adequacy.

Figure 3 shows that, of all the diseases studied in sample 2, severe asphyxia (but not all of the grades of this disease), hypoglycemia, hypomagnesemia, physiological jaundice, pulmonary hypertension, respiratory distress syndrome, congenital pneumonia and septicemia were shown to be associated with adequacy (p < 0.05). The most frequent diseases among the "small for length" infants were physiological jaundice, hypoglycemia and severe asphyxia, as can be seen in Figure 3.

**Figure 4:**
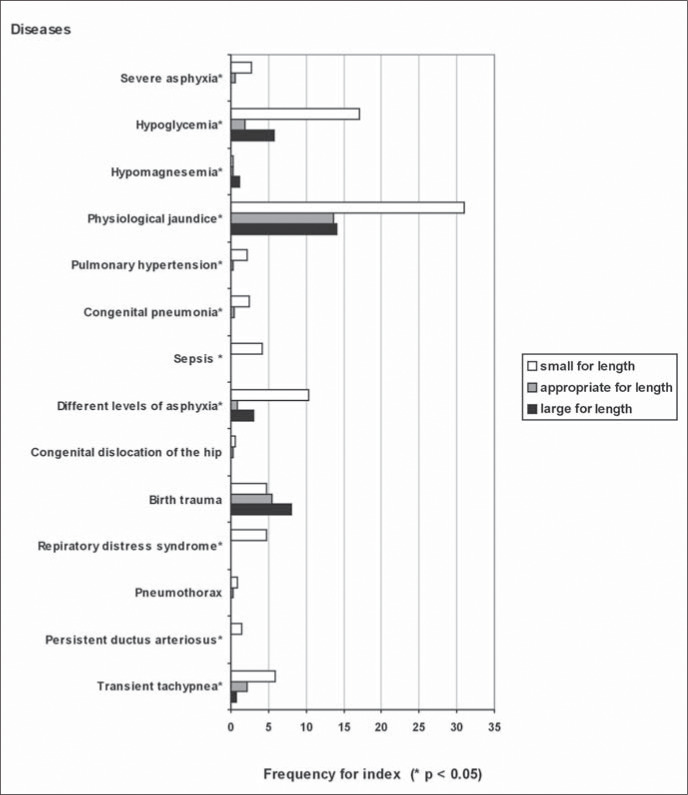
Neonatal disease frequency for index (W/L).

Figure 4 shows the relationships between the weight-for-length index distribution and the selected neonatal diseases that were studied in sample 2. All diseases, except for congenital dislocation of the hip, birth trauma and pneumothorax, showed an association with the index, as proven by p < 0.05 values. The associated diseases were more frequent in the "small index-for-length" category, except for hypomagnesemia, which was more frequent among infants that were "large index-for-length", and hypoglycemia, which was seen to be more frequent in both the small and large categories.

## DISCUSSION

The evaluation of newborns according to weight and length parameters is usually accomplished by using the ponderal index or body mass index.^11^ The simple relationship between weight and length has been used for comparing growth among children over a certain period of time^4,12^ but it is not usually used for investigating the association with neonatal diseases. For this reason, the present paper deals with this matter.

The newborn weight and length data taken from our population of high socioeconomic level were subjected to various methods of analysis. It was noted that weight and length variables were positively and almost linearly correlated, thereby allowing the establishment of the weight-forlength adequacy and its 10^th^ and 90^th^ percentiles and thus identifying newborns as "small-for-length", "appropriate-for-length" and "large-for-length".

The 10^th^ and 90^th^ percentiles were also calculated with regard to the weight-for-length index, thereby allowing newborns below the 10^th^ percentile to be classified as having "small index-for-length", those between the 10^th^ and 90^th^ percentiles (inclusive) as "appropriate index-for-length" and those above the 90^th^ percentile as "large index-for-length".

The relationship between gestational age and weight-for-length adequacy showed no differences in relation to preterm newborns in the three adequacy categories (small, appropriate and large for length). Conversely, there was a significant difference between the preterm newborn frequency in the three index categories, with the highest frequency noted for "small index-for-length" and the lowest for "large index-for-length". This difference in behavior can be explained on the basis of how these parameters were designed. The weight-for-length adequacy classification subdivided the sample into three categories: small, appropriate and large for each length class. Length increases as gestational age in-creases,^5^ but for each length there was a weight stratification in the sample. Since length is correlated with gestational age, it can be seen that there was an indirect correlation with the interference of the gestational age.

There was a higher preterm frequency for "small index-for-length" than for the "appropriate" and "large" categories. Since fatty deposits and muscle mass increase as pregnancy progresses,^7^ the weight/length quotient is lower for preterm than for full term infants. This suggests not only that undernourished full term newborns will be found in the "small index-for-length" category, but also preterm newborns that are not necessarily undernourished. Within the "large index-for-length" category there were more full term newborns and fewer preterm newborns. In evaluating the Kramer et al.^13^ paper on restriction of intrauterine growth, it was possible to calculate from their data how many infants in the "small index-for-length" category the authors would have had. It was seen that newborns considered to be undernourished would also be classified as such by the weight-for-length index established in the present study.

Sample 2 was described according to the same characteristics as sample 1. Over the whole study period, no changes took place in the Institution that could have interfered in the composition of the population attended. However, the two populations differed in terms of maternal age and newborn weight. Concerning maternal age, Duccini dal Colletto et al.^14^ demonstrated a rising trend from 1995 to 1998 when studying data from the same hospital.

When analyzing associations between diseases and the two methods of correlating weight to length, it was taken into account that adequacy evaluates body proportions without being influenced by gestational age, whereas the index is influenced by gestational age. It was thus noted that some diseases correlated via both methods, some only to the weight-for-length index and others did not correlate to either of the methods.

Thus, severe asphyxia, hypoglycemia, physiological jaundice, pulmonary hypertension, congenital pneumonia and sepsis showed a similar tendency to be more frequent within the "small weight-for-length" category. In all of these diseases, their association with intrauterine restriction can explain this relationship. In 1990, Villar et al.^15^ found a relationship between ponderal index below the 10^th^ percentile and severe asphyxia, meconium aspiration syndrome, hypoglycemia and infection. In calculating a weight-for-length index based on the data gathered by these authors, it was found that the same relationship would be found for the weight-for-length index below the 10^th^ percentile.

With regard to respiratory distress syndrome, different levels of asphyxia, persistent ductus arteriosus and transient tachypnea, the association was noted only for the index and the greatest incidence was noted among the "small indexfor-length" newborns. Therefore, one can speculate that prematurity was significantly important in the genesis of these diseases. The "small index-for-length" category represented not only the undernourished full term newborns but also the preterm newborns and probably all those of very low birth weight, where almost all disease occurrences are concentrated.^16^ In 1997, Williams and O'Brien,^1^ concluded that below the 10^th^ percentile the simple weight for length correlation was associated not only with perinatal depression, in accordance with the data of this study, but also with dysmaturity, cerebral palsy and higher mortality.

Hypomagnesemia has been shown to be associated both with adequacy and index, with higher incidence occurring within the "largefor-length" and "large index-for-length" categories. Among our samples, there was probably no occurrence in preterm newborns due to preventive measures routinely taken in the neonatal unit, with early administration of calcium and magnesium via the hydration serum given to these children.

Birth trauma, congenital dislocation of the hip and pneumothorax did not show an association with either adequacy or index.

## Conclusions

The almost linear correlation between weight and length allowed the construction of a curve considering only the two parameters of weight and length, as well as the identification of the 10^th^ and 90^th^ percentiles, independent of knowledge of the gestational age.

The classification of newborns into small, appropriate and large for length, or into small, appropriate or large index-for-length, allowed the identification of children at risk for some neonatal diseases. It is not necessary to know the gestational age for either classification and they are independent of each other. The same newborns may therefore be identified with regard to their risk for diseases either by adequacy or the weight-for-length index.

## FINAL REMARKS

Adequacy represents factors related to certain neonatal diseases and one can speculate that adequacy could possibly help in clarifying their pathophysiological origins. The weight-for-length index represents factors associated not only with intrauterine growth restriction but also with prematurity. As the weight-for-length index proved to be more discriminating, its application would therefore seem to be more useful.

Although the results presented here may contribute towards identifying some diseases occurring during the neonatal period for a population similar to the one presented in this study, it is recommended that each neonatology unit should know its population distribution according to weight and length so as to have an extra parameter available, which could be extremely useful in identifying risks for their specific patients.
